# Diet, Microbiome and Depression: Neuroscientific Perspectives

**DOI:** 10.1192/j.eurpsy.2026.11442

**Published:** 2026-07-31

**Authors:** M. Arts, M. Zeilstra, K. M. Zeilstra-Kalt, L. D. Jonge

**Affiliations:** 1 Geriatric Psychiatry and Neuropsychiatry, GGZ WNB, Halsteren; 2 Integrative Medicine, Center de Wetering, Leeuwarden; 3 Integrative Medicine, Leonardo Scientific Research Institute, Groningen, Netherlands

## Abstract

**Introduction:**

Depression is a leading cause of disability worldwide, and current pharmacological approaches are often insufficient. Increasing evidence suggests that the gut microbiome, through its bidirectional communication with the central nervous system, plays a crucial role in mood regulation and cognitive functions. Diet emerges as a modifiable factor influencing this brain-gut axis, thereby offering novel preventive and therapeutic opportunities.

**Objectives:**

This review aims to (a) describe the main mechanisms linking gut microbiota and brain function, (b) evaluate the role of dietary patterns and psychobiotics in modulating depression, and (c) assess the clinical potential of microbiota-based nutritional interventions.

**Methods:**

In this study, a literature review was conducted, synthesizing findings from microbiology, nutrition, and neuroscience. Sources included preclinical models, randomized controlled trials and clinical studies investigating probiotics, prebiotics, and dietary interventions in depressive disorders.

**Results:**

- Key pathways include microbial metabolites (short-chain fatty acids), tryptophan–kynurenine metabolism, vagal signaling, the hypothalamic-pituitary-adrenal (HPA) axis, and brain-derived neurotrophic factor (BDNF).
- Western diets are associated with pro-inflammatory microbiota profiles and reduced microbial diversity, while Mediterranean and MIND (combination of Mediterranean diet and dietary approaches to stop hypertension) diets correlate with greater diversity and lower depression risk. Clinical trials demonstrated significant symptom reduction following dietary modification.
- Altered microbial signatures in depression (e.g., reduced Bifidobacterium and Lactobacillus, increased Proteobacteria) were reported; some probiotic interventions improved mood outcomes.
- Nutrients such as omega-3 fatty acids, vitamin C, polyphenols, and folate appear to modulate stress response, neuroplasticity, and neurotransmission. Evidence from the PREDIMED trial linked Mediterranean diet with nuts to increased plasma BDNF levels after long-term follow-up.

**Conclusions:**

Gut microbiota-targeted nutritional strategies represent a promising adjunctive pathway for depression prevention and treatment. While mechanistic evidence is strong, clinical application remains limited by heterogeneity in microbiome composition, variability in interventions, and a predominance of small-scale or preclinical studies. Future large, standardized trials are essential to confirm efficacy and enable personalized lifestyle psychiatry approaches as complementary or alternative options to pharmacotherapy.

**Disclosure of Interest:**

None Declared

